# The p53 isoform delta133p53ß regulates cancer cell apoptosis in a RhoB-dependent manner

**DOI:** 10.1371/journal.pone.0172125

**Published:** 2017-02-17

**Authors:** Nikola Arsic, Alexandre Ho-Pun-Cheung, Crapez Evelyne, Eric Assenat, Marta Jarlier, Christelle Anguille, Manon Colard, Mikaël Pezet, Pierre Roux, Gilles Gadea

**Affiliations:** 1 CNRS, Centre de Recherche en Biologie cellulaire de Montpellier, Montpellier, France; 2 Université Montpellier, Montpellier, France; 3 Translational Research Unit, Institut du Cancer de Montpellier, Montpellier, France; 4 Department of Gastroenterology, Institut du Cancer de Montpellier, Montpellier, France; 5 Biostatistics Department, Institut du Cancer de Montpellier, Montpellier, France; 6 INSERM, Montpellier, France; 7 Université de la Réunion, Unité Mixte 134 Processus Infectieux en Milieu Insulaire Tropical, INSERM Unité 1187, CNRS Unité Mixte de Recherche 9192, IRD Unité Mixte de Recherche 249. Plateforme Technologique CYROI, Sainte Clotilde, France; Virginia Commonwealth University, UNITED STATES

## Abstract

The *TP53* gene plays essential roles in cancer. Conventionally, wild type (WT) p53 is thought to prevent cancer development and metastasis formation, while mutant p53 has transforming abilities. However, clinical studies failed to establish p53 mutation status as an unequivocal predictive or prognostic factor of cancer progression. The recent discovery of p53 isoforms that can differentially regulate cell cycle arrest and apoptosis suggests that their expression, rather than p53 mutations, could be a more clinically relevant biomarker in patients with cancer. In this study, we show that the p53 isoform delta133p53ß is involved in regulating the apoptotic response in colorectal cancer cell lines. We first demonstrate delta133p53ß association with the small GTPase RhoB, a well-described anti-apoptotic protein. We then show that, by inhibiting RhoB activity, delta133p53ß protects cells from camptothecin-induced apoptosis. Moreover, we found that high delta133p53 mRNA expression levels are correlated with higher risk of recurrence in a series of patients with locally advanced rectal cancer (n = 36). Our findings describe how a WT *TP53* isoform can act as an oncogene and add a new layer to the already complex p53 signaling network.

## Introduction

The *TP53* tumor suppressor gene regulates many physiological cellular processes. In response to stress, p53 is rapidly activated to promote cell cycle arrest, DNA repair and apoptosis [1]. This pro-apoptotic function is a key component of p53 tumor suppressor activity [2,3]. p53-stimulated apoptosis involves disruption of the mitochondrial membrane potential, accumulation of reactive oxygen species, stimulation of caspase 9 activity and the subsequent activation of the caspase cascade [4,5,6]. In cancer cells, p53 function is frequently altered due to *TP53* gene mutations or defects in p53 regulation and signaling. Consequently, p53 pro-apoptotic activity also is affected, allowing cancer cells to progress towards a more aggressive phenotype. However, in the clinic, it is difficult to link p53 mutation status with cancer progression, prognosis or response to treatment, suggesting that other regulatory mechanisms influence the p53 tumor suppressor pathway [7,8,9,10].

The human *TP53* gene encodes at least twelve p53 isoforms through alternative splicing of intron-2 (delta40) and intron-9 (α, ß and γ), alternative promoter use (delta133) and alternative initiation of translation at codon 40 (delta40) and codon 160 (delta160) [11]. p53 isoforms can modulate p53 activity and have different effects on cell fate by differentially regulating cell cycle arrest, replicative senescence and apoptosis [11]. Furthermore, p53 isoforms are abnormally expressed in many tumors, including breast and colon cancers, suggesting that they could play a role in cancer formation and progression (for review [12]). The delta133p53 mRNA variants encode three short p53 isoforms: delta133p53α, delta133p53ß and delta133p53γ [11,13]. These isoforms lack the N-terminal transactivation domains and part of the DNA-binding domain. In analyzing a cohort of breast cancer patients, we recently showed that delta133p53ß expression is strongly correlated with metastatic dissemination and patients’ death. Moreover, this study indicated that delta133p53ß facilitates spreading to other organs of breast cancer cells that express wild type (WT) or mutated *TP53* gene [14]. Delta133p53ß enhanced migration and invasion, and promotes EMT in a panel of breast cancer cells. The same mechanism happened in colon cancer cells [14]. We also showed that delta133p53ß promotes *in vitro* and *in vivo* cancer stem cell (CSC) potential [15], a feature which is acquired by metastatic cells and which confers resistance to therapeutic regiment by modifying apoptosis response. It was also demonstrated that the zebrafish homologue of human delta133p53 antagonizes p53 apoptotic activity by specifically upregulating anti-apoptotic gene expression [16]. This suggests that N-terminally truncated p53 isoforms could negatively modulate p53 pro-apoptotic activity.

In this study, we investigated whether delta133p53 isoforms, and particularly the ß variant, could be involved in regulating the apoptotic responses of colorectal cancer (CRC) cell lines. We found that delta133p53ß directly binds to RhoB, a small GTPase with a well-described anti-apoptotic role. Furthermore, we showed that delta133p53ß inhibits RhoB tumor suppressor activity, thereby protecting tumor cells from RhoB-induced apoptosis. Finally, we assessed the prognostic value of delta133p53 expression in a series of 36 patients with locally advanced rectal cancer and found that delta133p53 mRNA expression quantification could be useful for identifying patients at risk of developing metastases. This study provides new insights into p53 isoform functions and their role in tumor progression.

## Results

### Delta133p53ß interacts with RhoB

**Fig 1:**
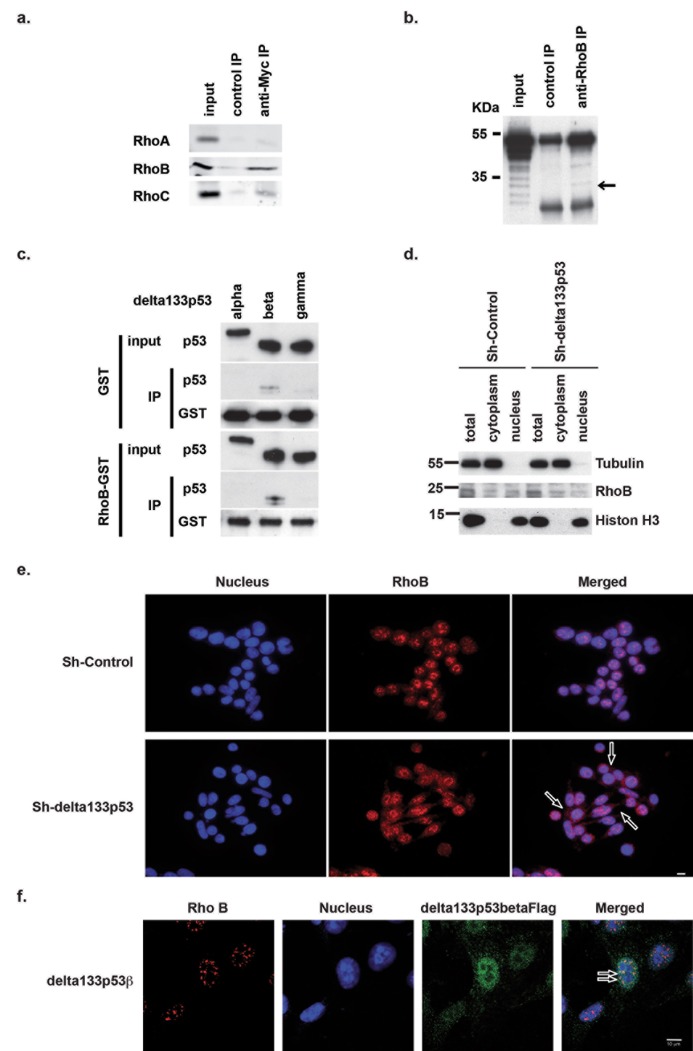
Delta133p53ß physically interacts with RhoB. (a) Immunoblot analysis showing the specific co-immunoprecipitation of MYC-tagged delta133p53ß and endogenous RhoB or RhoC, to a lower extent, but not RhoA. (b) Immunoblot analysis showing the co-immunoprecipitation of endogenous RhoB and delta133p53ß with anti-RhoB antibodies. Arrow shows co-immunoprecipitation of delta133p53ß isoform. (c) *In-vitro* binding assay showing the direct interaction between recombinant RhoB-GST fusion protein and delta133p53ß, but not α or γ. (d) Cellular fractionation showing RhoB cytoplasmic re-localization in SW620 cells transfected with shRNAS against delta133p53 compared with shControl (mock-transfected cells). (e) Immunofluorescence analysis of RhoB localization in control SW620 cells (shControl) or after transfection with shdelta133p53. Arrows indicate examples of RhoB cytoplasmic localization. Scale bar: 10μm. (f) Confocal images showing the co-localization of RhoB and delta133p53ß in the nucleus of SW480 cells that overexpress delta133p53ß. Arrows indicate examples co-localization of RhoB and delta133p53ß in the nucleus. Scale bar: 10μm.

In previous studies, we demonstrated that p53 regulates the activities of the small GTPases RhoA and CDC42 [17,18,19]. In parallel, many other publications focused on the small isoforms encoded by the *TP53* gene. Several of them modify p53 activities, including N-terminally deleted isoforms that in zebrafish modulate p53 apoptotic response [20]. As some small GTPases, particularly RhoB, are also involved in apoptotic signaling, we hypothesized that the N-terminally deleted human delta133p53 isoforms could have a role in apoptosis regulation through this GTPase. To test this hypothesis, we used a panel of CRC cell lines in which we previously characterized the expression of various p53 isoforms [14]. The HCT116 (WT *TP53*) and SW480 (mutant p53R273H) cell lines were derived from primary CRC and weakly express delta133p53 isoforms. On the other hand, the LoVo (WT *TP53*), SW620 (mutant p53R273H) and CoLo205 (mutant p53, Y103 del27bp) cell lines were generated from metastatic CRC and strongly express delta133p53 isoforms. First, we investigated whether delta133p53 isoforms interact with different small Rho GTPases. Co-immunoprecipitation experiments demonstrated that delta133p53ß specifically bound to RhoB and, to a lower extent, to RhoC, but not to RhoA (Fig 1A). Further co-immunoprecipitation analyses performed in HCT116 cells that overexpress delta133p53ß confirmed the interaction with RhoB (S1A Fig). These data were strengthened by co-immunoprecipitation experiments performed with endogenous proteins (Fig 1B). Besides delta133p53ß, additional p53 isoforms were co-immunoprecipitated with endogenous RhoB (upper bands in Fig 1B), most probably other isoforms with a ß C-terminal motif. These data indicate that endogenous delta133p53ß and RhoB are specifically associated in the same complex.

To assess whether delta133p53ß interacts directly with RhoB, we then performed *in vitro* binding assays in which GST-RhoB fusion protein was incubated with histidine-tagged delta133p53β, ß or γ. These experiments showed that only delta133p53ß could directly interact with RhoB (Fig 1C). Furthermore, depletion of delta133p53 isoforms using specific shRNAs resulted in RhoB delocalization from the nucleus to the cytoplasm (Fig 1D). This suggests that interaction with delta133p53ß sequesters RhoB in the nucleus. Similarly, immunodetection of RhoB in SW620 cells transfected or not with the indicated shRNAs (Fig 1E) showed that RhoB was predominantly nuclear in control cells (shControl), whereas it was delocalized to the cytoplasm following delta133p53 depletion (shdelta133p53). These data reinforce the hypothesis that interaction with delta133p53ß sequesters RhoB in the nucleus. Finally, co-localization analysis of RhoB and delta133p53ß by confocal microscopy (delta133p53α overexpression as control in S1C Fig) demonstrated that the two proteins co-localized in the nucleus (Fig 1F). Altogether, these data indicate that delta133p53ß specifically and directly interacts with RhoB to control its nuclear localization.

### Delta133p53ß negatively regulates RhoB activity

**Fig 2:**
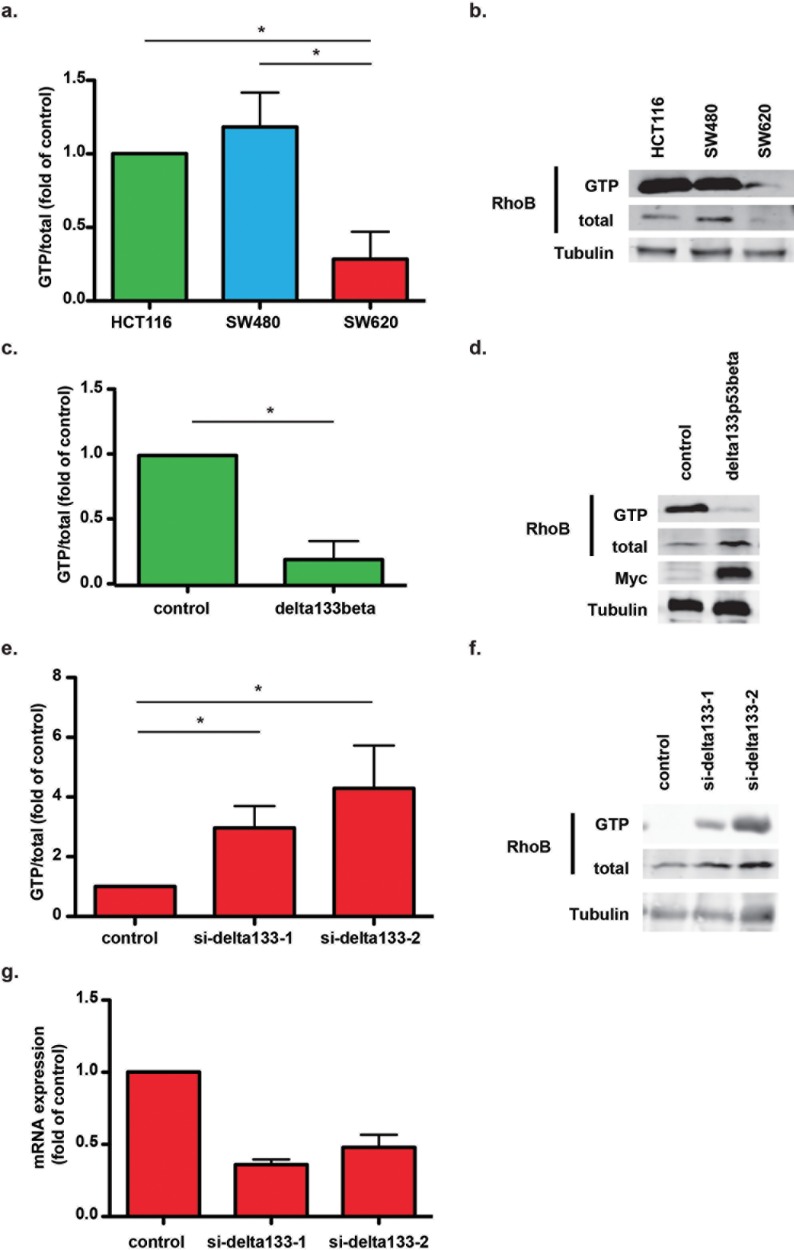
Delta133p53ß controls RhoB activity in CRC cell lines. (a) RhoB activity in HCT116, SW480 and SW620 cells was assessed by using GTPase activity assays. Results are expressed as the fold change compared with HCT116 cells and represent the mean ± SEM of four independent experiments; *, p<0.05. (b) Representative immunoblot showing levels of total and GTP-bound RhoB in HCT116, SW480 and SW620 cells. (c) RhoB activity in mock-transfected (control) and delta133p53ß-overexpressing HCT116 cells. Results are expressed as the fold change compared with control and represent the mean ± SEM of three independent experiments; *, p<0.05. (d) Representative immunoblot showing MYC-tagged delta133p53ß expression in delta133p53ß-overexpressing and mock-transfected (control) HCT116 cells. (e) RhoB activity in mock-transfected (control) SW620 cells and following transfection with two different siRNAs against delta133p53. Results are expressed as the fold change compared with control cells and represent the mean ± SEM of four independent experiments; *, p<0.05. (f) Representative immunoblot showing the levels of total and GTP-bound RhoB in control and delta133p53-depleted SW620 cells. (g) Depletion of delta133p53 isoforms in SW620 cells after siRNA transfection was assessed by RT-quantitative PCR. Results are expressed as the fold change compared with mock-transfected SW620 cells (control) and represent the mean ± SEM of four independent experiments.

Next, we asked whether the direct and specific interaction of delta133p53ß with RhoB could modulate RhoB GTPase activity. First, we assessed RhoB activity in the CRC cell lines HCT116, SW480 and SW620 (Fig 2A and 2B). RhoB activity was significantly lower in SW620 cells (derived from a CRC metastasis and with strong delta133p53 expression) compared with HCT116 and SW480 cells (originating from primary CRC samples and with weaker delta133p53 expression). Conversely, RhoC activity did not vary in the three cell lines (S1B Fig). We then evaluated whether RhoB activity changed following modifications in delta133p53ß expression. To this aim, we first measured RhoB activity in HCT116 cells that overexpress MYC-tagged delta133p53ß and found that RhoB activity was strongly decreased in overexpressing cells compared with mock-transfected control cells (Fig 2C and 2D). Conversely, depletion of delta133p53 isoforms by using two independent siRNAs clearly rescued RhoB activity in SW620 cells (Fig 2E, 2F and 2G). Altogether, these data indicate that delta133p53ß negatively regulates RhoB activity.

### Delta133p53ß protects cancer cell from apoptosis

**Fig 3:**
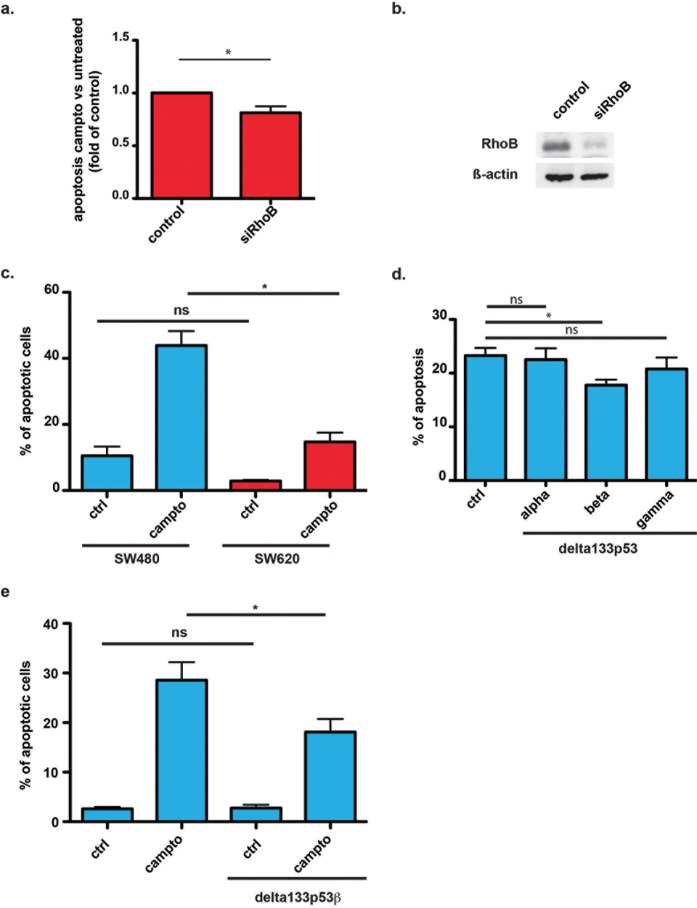
Delta133p53ß protects cancer cells from camptothecin-induced apoptosis. (a) Apoptosis assay showing the sensitivity to 5μM camptothecin of mock-transfected (control) SW620 cells and after transfection with siRNAs against RhoB. Results are expressed as the apoptosis ratio of untreated versus camptothecin-treated cells and represent the mean ± SEM of four independent experiments; *, p<0.05. (b) Immunoblot showing RhoB downregulation in siRhoB-transfected SW620 cells compared with mock-transfected cells (control). (c) Apoptosis assay to test the sensitivity of SW480 and SW620 cells to 5μM camptothecin. Results are expressed as the percentage of apoptotic cells relative to all Hoechst-positive cells and represent the mean ± SEM of three independent experiments; *, p<0.05. (d) Apoptosis assay showing the sensitivity to 5μM camptothecin of mock-transfected (control) and SW480 cells that stably express delta133p53α, ß or γ. Results are expressed as the percentage of apoptotic cells relative to all Hoechst-positive cells and represent the mean ± SEM of three independent experiments; *, p<0.05. (e) Apoptosis assay showing the sensitivity to 5μM camptothecin of mock-transfected and SW480 cells that stably express delta133p53ß. Results are expressed as the percentage of apoptotic cells relative to all Hoechst-positive cells and represent the mean ± SEM of four independent experiments; *, p<0.05.

As RhoB is considered a tumor suppressor due to its pro-apoptotic role [21], we investigated whether RhoB interaction with delta133p53ß affected apoptosis. To this aim, we used the chemical compound camptothecin that induces apoptosis in many normal and tumor cell lines and also promotes RhoB expression and RhoB-induced apoptosis [22]. Moreover, irinotecan, a synthetic analog of camptothecin, is routinely used for the treatment of patients with CRC. To confirm that RhoB is involved in camptothecin-induced apoptosis in our cell system, we investigated the effect of RhoB knockdown in SW620 cells. RhoB depletion with specific siRNAs reduced camptothecin-induced apoptosis in CRC cells (Fig 3A and 3B). Then, we compared the apoptosis rate in SW480 (low delta133p53ß expression/high RhoB activity) and SW620 (high delta133p53ß expression/low RhoB activity) cells after incubation with camptothecin. SW480 cells were more sensitive to camptothecin than SW620 cells, whereas the apoptosis rates of untreated cells were not significantly different (Fig 3C). To confirm that delta133p53 is involved in reducing the apoptotic effect of RhoB, we tested the sensitivity to camptothecin-induced apoptosis of SW480 cell lines that stably overexpress delta133p53ß, delta133α or γ (Fig 3D). As expected, delta133p53ß-overexpressing SW480 cells were less sensitive to camptothecin than the parental cell line, but not cells overexpressing delta133p53α or γ. This is in agreement with the observation that only delta133p53ß interacts with RhoB. Finally, we compared the level of anti-apoptotic protection in SW480 cells that overexpress or not delta133p53ß. SW480 cells that overexpress this isoform were significantly less sensitive to camptothecin-induced apoptosis than control (mock-transfected) cells (Fig 3E). Altogether these findings demonstrate that only delta133p53ß can modulate RhoB activity and protect CRC cells from camptothecin-induced apoptosis.

### Delta133p53 isoforms are potential prognostic biomarkers in colorectal cancers

The finding that delta133p53ß can protect cells from apoptosis suggests that tumors with high delta133p53ß expression levels might be less responsive to therapy. Moreover, Gong et al. demonstrated that delta133p53 is strongly induced by ionizing radiation and protects cells from death by preventing apoptosis and promoting DNA double strand break repair [23]. This suggests that high expression of delta133p53 isoforms could be associated with tumor resistance.

**Fig 4:**
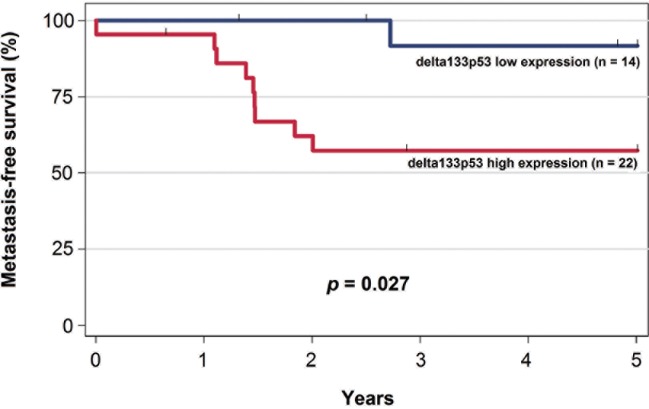
Kaplan–Meier curves of metastasis-free survival in the cohort of 36 patients with rectal cancer stratified according to delta133p53 expression (low/high).

We thus asked whether delta133p53 expression could be a potential prognostic factor in patients with CRC. To this aim, we assessed delta133p53 mRNA expression levels in pre-treatment endoscopic biopsies from a cohort of 36 patients with locally advanced rectal cancer who received standard preoperative radio-chemotherapy (Table 1). We then dichotomized patients according to the expression level of the delta133p53 isoforms in low and high expression groups using a cut-off value derived from the ROC curve for predicting distant metastases (see also Materials and Methods). The selected cut-off value corresponded to the most appropriate threshold to maximize both sensitivity and specificity for the prediction of distant metastases. After a median follow-up of 5.1 years (range: 1.7–7.3 years), 10 patients (27.8%) had distant metastases. No correlation was found between delta133p53 expression and clinical parameters, such as age, gender, tumor grade and pre-treatment Union for International Cancer Control (UICC) Tumor-Node-Metastasis (TNM) staging (data not shown). On the other hand, high delta133p53 expression levels (Fig 4) were significantly correlated with higher risk of metastatic recurrence (p = 0.027; log-rank test). This suggests that delta133p53 plays a major role in cancer progression and could be used as a prognostic biomarker to assess the clinical outcome.

**Table 1:** Patients’ characteristics.

Characteristics	No of patients (%)
**Age, years**	
Median	61
Range	42–82
**Gender**	
Male	28 (77.8)
Female	8 (22.2)
**Pre-treatment UICC TNM stage**	
Stage II	8 (22.2)
Stage III	28 (77.8)
**Tumor Grade**	
Well Differentiated	12 (33.3)
Moderately Differentiated	21 (8.3)
Poorly Differentiated	3 (58.4)
**Postoperative treatment**	
No	24 (66.7)
Yes	12 (33.3)

Abbreviations: TNM, Tumor, Node, and Metastasis; UICC, International Union Against Cancer.

## Discussion

Several previous works, including some from our group, have described the functional interaction between p53 pathways and actin cytoskeleton regulators through modulation of small Rho GTPase activity [17,18,24]. This study is the first to demonstrate the direct role of a p53 isoform in regulating the activity of a Rho GTPase. RhoA, B and C, members of the Rho group of GTPases, have very well- described roles in cancer progression. While RhoA and C promote invasion and metastasis formation through regulation of actin cytoskeleton dynamics, several findings suggest that RhoB has a tumor suppressor activity through its pro-apoptotic function [25,26,27,28,29,30]. Moreover, RhoB expression can inhibit metastasis formation [31,32]. Our study clearly demonstrates that the delta133p53ß isoform interacts with RhoB, and to a lower extent with RhoC, but not with RhoA. As RhoA, B and C share a high level homology, RhoB specific interaction with delta133p53ß could be explained by the different cellular localization of these GTPases. Indeed, RhoB is the only farnesylated Rho GTPase and this leads to a different localization compared with RhoA and C. We also found that only the ß isoform interacts with RhoB, indicating that its C-terminus, which is different from that of the other delta133p53 isoforms, is responsible for this binding. This could also explain the presence of additional p53 isoforms, possibly delta40p53ß, after co-immunoprecipitation of endogenous RhoB and p53 isoforms. A more complete structure/function analysis is now required to clarify this point.

The *TP53* gene exercises its tumor suppressive functions mainly by inducing cell cycle arrest and apoptosis. Our data show that *TP53*, through expression of the delta133p53ß isoform, can inhibit apoptosis and *de facto* acts as an oncogene. As such, delta133p53ß represents a new oncogene that controls RhoB tumor suppressive function. Furthermore, our data suggest a possible mechanism of action for delta133p53ß through RhoB retention in the nucleus. Our findings also demonstrate the existence of an active mechanism of RhoB action inhibition by delta133p53ß because we observed reduction of RhoB exchange activity in the presence of this isoform. Based on our results, we propose a model of RhoB pro-apoptotic activity regulation by delta133p53ß through modulation of its cellular localization and GTPase exchange activity (S2 Fig).

The finding that delta133p53ß can protect cells from camptothecin-induced apoptosis may have a clinical impact. Indeed, camptothecin analogs have been approved and are currently used for cancer treatment. Particularly, irinotecan, a synthetic analog of camptothecin, is a key component of chemotherapy regimens for CRC [33] in both adjuvant and metastatic settings. Moreover, our clinical data demonstrate a clear correlation between high delta133p53 expression level and rectal cancer recurrence, thus strengthening the possible role of this isoform in cancer progression through suppression of RhoB-induced apoptosis. Distant recurrences are observed in 24–36% of patients with rectal cancer [34] and are associated with high morbidity and mortality. Hence, the identification of patients at high risk of distant metastases represents a major challenge. The present data suggest that quantification of delta133p53 mRNA expression in pre-treatment biopsies could help identifying patients at risk of developing metastases. In conclusion, this study identified delta133p53ß as a potential biomarker of patients with rectal cancer at high risk of metastatic disease.

## Materials and methods

### Cells and reagents

The CRC cell lines HCT116, SW480, LoVo, SW620 and Colo205 were purchased from ATCC and cultured as recommended. Cells were transfected for 24 hours with plasmids using the jetPEI reagent (Polyplus) according to the manufacturer’s recommendations. For stable expression experiments, cells transfected with MSCV plasmids were selected with 200 μg/mL hygromycin B (Life Technologies) for 2 weeks. Resistant colonies were pooled and delta133p53ß overexpression was confirmed by immunoblotting (not shown). For silencing experiments, cells were transfected with two sh/siRNAs targeting the delta133p53 isoforms (sh/si-delta133-1: CUUGUGCCCUGACUUUCAA and sh/si-delta133-2: GGAGGUGCUUACACAUGUU) using the INTERFERin reagent (Polyplus) according to the manufacturer’s recommendations, 72 hours prior to RhoB activity testing.

### Reverse transcription, nested PCR, quantitative PCR

Total RNA was extracted from cells (Qiagen) and reverse transcription performed with 1 μg of total RNA, oligo d(T) primer and SuperScript® III Reverse Transcriptase (Life Technologies) at 50°C for 50 minutes. cDNA was then amplified by two consecutive PCR reactions (nested PCR) of 30 cycles/each with PCR primers specific for each of the p53 isoforms analyzed. The primer sequences have been previously described [11,35]. Quantification of delta133p53 was performed by TaqMan real-time PCR on a LightCycler480 apparatus (Roche). Briefly, 20 ng cDNA was amplified using 0.8 μM of each primer, 0.4 μM of probe (see S3 Fig) and 1X LightCycler® 480 Probes Master Mix (Roche). All data were normalized to the internal standard TBP. For each single-well amplification reaction, a threshold cycle (Ct) was calculated using the LightCycler480 program (Roche) in the exponential phase of amplification. Relative changes in gene expression were determined using the 2ΔΔCt method and reported relative to control.

### GTPase activity assay

GTPase activity assays were performed as described in [36]. Briefly, 3.10^6^ cells were lysed before incubation with the GST-RBD fusion protein (RBD is the Rho-binding domain of human Rhotekin; amino acids 7–89) to assess RhoB/C activity, coupled to Glutathione–Sepharose beads (Cytoskeleton). After precipitation, complexes were immunoblotted. Aliquots taken from supernatants prior to precipitation were used to quantify the total GTPases present in cell lysates. Quantifications were done with the Odyssey Infrared Technology (LI-COR).

### Immunoblotting

Protein samples were resolved on 12% SDS-PAGE gels and analyzed with antibodies against RhoB (Santa Cruz Technologies, sc-180), α-tubulin (Sigma, T6199), MYC (Santa Cruz Technologies, sc-789), RhoC (Cell Signaling Technology, clone D40E4), p53 (mAb 240 or SAPU, kind gifts of J.C. Bourdon), Flag (clone M2 from Sigma-Aldrich) and GST (kind gift of G. Bossis).

### Co-immunoprecipitation experiments

Cells were harvested in cell lysis buffer (10mM Tris-HCl pH 7.5, 150mM NaCl, 10mM MgCl_2_, 0.5% Triton X-100, 10mM dithiothreitol, protease/phosphatase inhibitor cocktails). Cell extracts were then incubated with monoclonal anti-RhoB antibodies (2 or 4 μg) and 50μl of anti-mouse IgG magnetic beads (Dynal) at 4°C for 4 hours. After washing (lysis buffer with 1% Triton X-100), bound proteins were eluted with SDS sample buffer and analyzed by immunoblotting.

### *In-vitro* binding assay

RhoBΔ18 cDNA was cloned in the pGSTII bacterial expressing vector (kind gift of K. Burridge). The delta133p53 α, ß and γ isoforms were expressed using the pET28a Histidine-tag vector. Recombinant proteins were produced in the B21 *E*. *coli* strain. GST and GST-RhoBΔ18 were purified using Glutathione Sepharose (GE Healthcare) according to the manufacturer’s instructions without elution from the beads. The delta133p53 α, ß and γ isoforms were extracted using Histidine Select Nickel Affinity Gel (Sigma), according to the manufacturer’s instructions. Eluted recombinant p53 isoforms were incubated on ice with GST or GST-RhoBΔ18 Glutathione Sepharose beads for 10 minutes. After washes, beads were resuspended in loading buffer and analyzed by immunoblotting.

### Cell fractionation

Cell fractionation was performed following the REAP method described in [37]. This method drastically reduces the time needed for subcellular fractionation, eliminates detectable protein degradation and maintains protein interactions. The simplicity, rapidity and efficiency of this procedure allow tracking ephemeral changes in subcellular localization of proteins, while maintaining protein integrity and protein complex interactions.

### Immunostaining

Anti-RhoB and/or anti-Flag primary antibodies and Alexa Fluor secondary antibodies (Life Technologies) were used. Cells were fixed in 3.2% paraformaldehyde and permeabilized with 0.2% Triton X-100. Nuclei were labeled with Hoechst (Sigma-Aldrich). After mounting with ProLong Gold anti-fade agent (Invitrogen, Molecular Probes), cells were visualized using a Zeiss Axioimager Z1 or a Leica SP5 confocal microscope coupled to the MetaMorph software (Molecular Devices).

### Apoptosis assay

10.000 cells/well were plated in 96-well plates and allowed to grow in 100μl of complete medium (10% fetal calf serum, 50 units/mL penicillin, 50 μg/mL streptomycin). After 24 hours, cells were incubated with 5 μM camptothecin (Sigma-Aldrich) for another 24 hours. Medium was then removed and replaced by NucView™ 488 Caspase-3 Substrate (Biotium) diluted as recommended by the manufacturer. NucView™ 488 Caspase-3 Substrate is a fluorescent probe that allows detecting caspase 3/7 activity in intact cells in real-time. After 15 minutes and without removing the NucView™ 488 reagent, cells were simultaneously fixed in 3.7% (final concentration) paraformaldehyde and stained with 1 μg/ml Hoechst (Sigma, B2261). After at least 2 hours of incubation at room temperature in the dark, plates were read using a Cellomics ArrayScan VTI HCS Reader (Thermo Scientific). Results were acquired and analyzed using the Target Activation BioApplication. Cells co-stained by Hoechst and NucView™ 488 Caspase-3 Substrate were scored as apoptotic. Each sample was assessed in quadruplicate and 16 fields per well were analyzed.

### Patient samples and statistical analysis

Our cohort consisted of 36 Caucasian patients (28 men and 8 women), with a median age of 61 years at the time of diagnosis (range: 42–82 years). Inclusion criteria were: 1) histologically confirmed diagnosis of sporadic rectal adenocarcinoma, 2) indication for neoadjuvant radiochemotherapy, and (3) absence of synchronous metastases.

This study was reviewed and approved by the Montpellier Cancer Institute Institutional Review Board (ID number ICM-URC-2013/58). Considering the retrospective, non-interventional nature of this study, no specific consent was deemed necessary by the Montpellier Cancer Institute clinical research review board. The original study [33,34] was approved by the French Ethics Committee for the protection of patients of Saint-Eloi Hospital (Montpellier, France), and declared in ClinicalTrials.gov (ID number NCT00628368). Written consent was obtained for each enrolled patient.

For all patients, four to six pre-treatment biopsies of the primary rectal adenocarcinoma were obtained by endoscopy, and RNA extraction was performed using the RNeasy Mini Kit (Qiagen) following the manufacturer’s instructions. To limit the inter-assay variation, all samples were reverse-transcribed simultaneously using the same reagents and master mix, as previously described [38]. Primers that matched a common sequence in delta133p53α, delta133p53ß and delta133p53γ mRNA were used to quantify the total expression of all delta133p53 mRNA variants. For accurate comparison of delta133p53 expression level in the different samples, RNA degradation-related variations were normalized using a previously described RNA integrity number (RIN)-based algorithm we developed [39]. Possible associations between delta133p53 mRNA levels and the patients’ clinical characteristics were assessed using the chi-square test, Fisher’s exact test or Wilcoxon rank sum test, as appropriate. Metastasis-free survival was calculated from the date of surgery to the date of diagnosis of distant metastases or of the last follow-up. Univariate survival analyses were then performed using the Kaplan–Meier method. For statistical purposes, patients (n = 36) were classified in two groups (high or low delta133p53 expression level; respectively 22 and 14 patients) according to a cut-off value derived from the receiver-operating-characteristic (ROC) curve for predicting distant metastases. A cut-off value that maximized the Youden’s index was selected. This index is defined as the sum of the sensitivity and specificity minus 1 and is frequently used to dichotomize continuous variables in ROC curve analyses. All statistical analyses were performed with STATA 11.0 (StataCorp, College Station, TX).

## Supporting information

S1 Fig Analysis of RhoB-delta133p53ß interaction and of RhoC activity in CRC cell lines and RhoB localization in delta133p53α-overexpressing SW480 cells. (a) Immunoblot showing the co-immunoprecipitation of MYC-tagged delta133p53ß and endogenous RhoB. (b) RhoC activity in HCT116, SW480 and SW620 cells. Results are expressed as the fold change compared with RhoC activity in HCT116 cells and represent the mean ± SEM of three independent experiments. (c) Confocal images showing the localization of RhoB and delta133p53α in delta133p53α-overexpressing SW480 cells. Scale bar: 10μm.
(JPG)

S2 Fig Schematic illustration of the role of delta133p53ß in regulating RhoB pro-apoptotic activity. When delta133p53ß is expressed in cancer cells, RhoB is sequestered in the nucleus and its activity is inhibited. In the absence of delta133p53ß, RhoB can trigger its pro-apoptotic activities in the cytoplasm.
(JPG)

S3 Fig Sequences of primers and probes used for TaqMan real-time PCR assays. (DOCX)
