# Correction: The p53 isoform delta133p53ß regulates cancer cell apoptosis in a RhoB-dependent manner

**DOI:** 10.1371/journal.pone.0175607

**Published:** 2017-04-06

**Authors:** Nikola Arsic, Alexandre Ho-Pun-Cheung, Evelyne Lopez-Crapez, Eric Assenat, Marta Jarlier, Christelle Anguille, Manon Colard, Mikaël Pezet, Pierre Roux, Gilles Gadea

The third author’s name appears incorrectly. The correct name is: Evelyne Lopez-Crapez. The correct citation is: Arsic N, Ho-Pun-Cheung A, Lopez-Crapez E, Assenat E, Jarlier M, Anguille C, et al. (2017) The p53 isoform delta133p53ß regulates cancer cell apoptosis in a RhoB-dependent manner. PLoS ONE 12(2): e0172125. doi:10.1371/journal.pone.0172125
