# Supplementary figures and images for: The p53 isoform delta133p53ß regulates cancer cell apoptosis in a RhoB-dependent manner
Source: PLoS One. 2017 Feb 17;12(2):e0172125. doi: 10.1371/journal.pone.0172125 (PMC5315499; doi:10.1371/journal.pone.0172125)

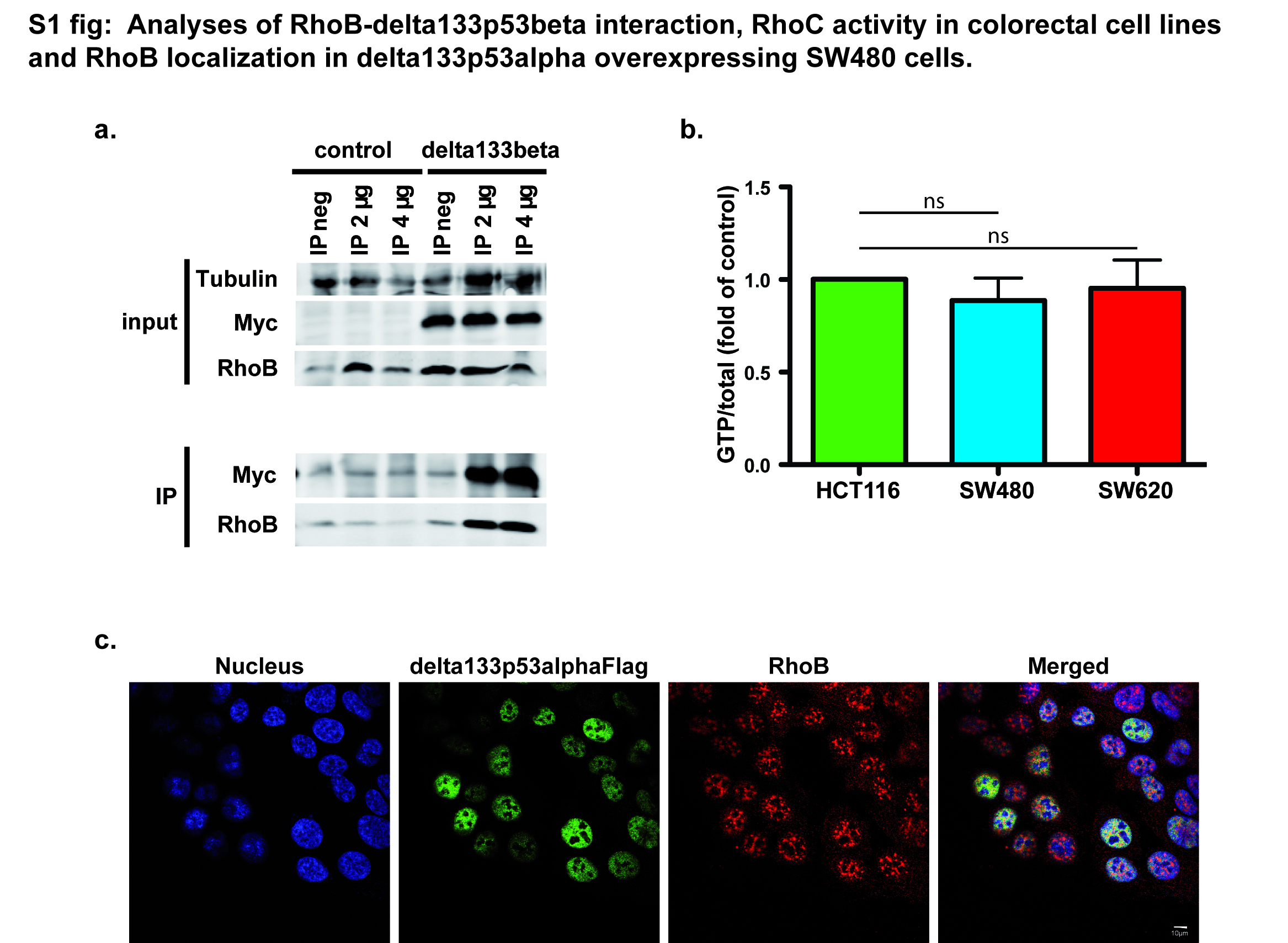

Supplement: S1 Fig — (a) Immunoblot showing the co-immunoprecipitation of MYC-tagged delta133p53ß and endogenous RhoB. (b) RhoC activity in HCT116, SW480 and SW620 cells. Results are expressed as the fold change compared with RhoC activity in HCT116 cells and represent the mean ± SEM of three independent experiments. (c) Confocal images showing the localization of RhoB and delta133p53α in delta133p53α-overexpressing SW480 cells. Scale bar: 10μm. (JPG) [file pone.0172125.s001.jpg]

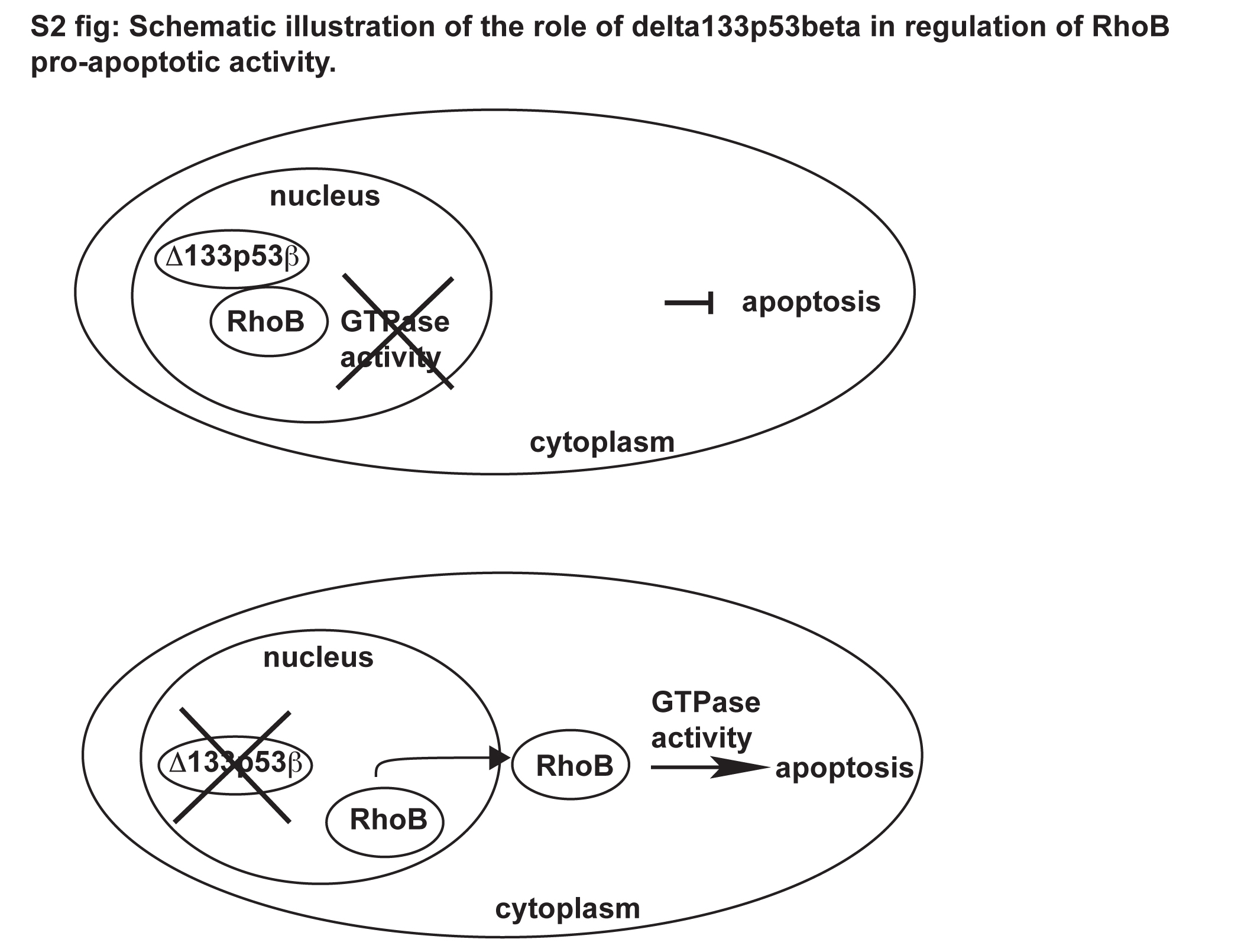

Supplement: S2 Fig — When delta133p53ß is expressed in cancer cells, RhoB is sequestered in the nucleus and its activity is inhibited. In the absence of delta133p53ß, RhoB can trigger its pro-apoptotic activities in the cytoplasm. (JPG) [file pone.0172125.s002.jpg]
