# Supplementary material for: The p53 isoform delta133p53ß regulates cancer cell apoptosis in a RhoB-dependent manner
Source: PLoS One. 2017 Feb 17;12(2):e0172125. doi: 10.1371/journal.pone.0172125 (PMC5315499; doi:10.1371/journal.pone.0172125)
Supplement: S3 Fig — (DOCX) [file pone.0172125.s003.docx]

**S3 Fig**: primers and probes sets for TaqMan quantitative PCR

delta133p53: targets all delta133p53 isoforms

TBP: housekeeping gene

| delta133p53 | Reverse | GTGTGGAATCAACCCACAGCT |
| --- | --- | --- |
|  | Probe | TCCCCTGCCCTCAACAAGATGTTTTGCC |
|  | Forward | ACTCTGTCTCCTTCCTCTTCCTACAG |
| TBP | Forward | CACGAACCACGGCACTGATT |
|  | Reverse | TTTTCTTGCTGCCAGTCTGGAC |
|  | Probe | TGTGCACAGGAGCCAAGAGTGAAGA |
